# Elucidation of the mechanism underlying impaired sensorimotor gating in patients with primary blepharospasm using prepulse inhibition

**DOI:** 10.3389/fneur.2023.1105483

**Published:** 2023-02-01

**Authors:** Xinqing Hao, Xiaofeng Huang, Xiaoxue Yin, Hai-Yang Wang, Ren Lu, Zhanhua Liang, Chunli Song

**Affiliations:** ^1^Department of Neurology, The First Affiliated Hospital of Dalian Medical University, Dalian, China; ^2^Department of Neurology, Jining No. 1 People's Hospital, Jining, China

**Keywords:** primary blepharospasm, prepulse inhibition, blink reflex, sensory trick, sensorimotor integration

## Abstract

**Objective:**

We aimed to analyze prepulse inhibition (PPI) impairment of the blink reflex in patients with primary blepharospasm (BSP).

**Methods:**

We recruited 30 BSP patients and 20 gender- and age-matched healthy controls (HCs). Weak electrical stimulation was applied to the right index finger at interstimulus intervals (ISIs) of 120, 200, and 300 ms before the supraorbital nerve stimulation to investigate PPI size [PPI size = (1 – R_2_ area at prepulse trials/R_2_ area at baseline trials) × 100%].

**Results:**

The prepulse stimulus significantly inhibited the *R*_2_ component at the three ISIs in both groups, but less inhibition was shown in the BSP group (*p* < 0.05). In HCs, the prepulse stimulus induced prolonged *R*_2_ and *R*_2c_ latencies at the three ISIs and increased the *R*_1_ amplitude at ISIs of 120 ms; these changes were absent in BSP patients. In the BSP group, patients with sensory tricks showed better PPI than patients without sensory tricks. Disease duration and motor symptom severity showed no significant correlation with PPI size.

**Conclusion:**

In BSP patients, PPI was impaired while *R*_1_ facilitation was absent. PPI size did not correlate with the motor symptom severity and disease duration. Patients with sensory tricks showed better PPI than those without sensory tricks.

## 1. Introduction

Primary blepharospasm (BSP) is a common focal dystonia disorder characterized by intermittent or persistent involuntary eye closure (1). Although recognized as a movement disorder, various studies have shown that abnormal sensory processing plays an important role in the pathophysiology of BSP (2). Local sensory symptoms, such as burning sensation in the eye, photophobia and dry eye, may precede the onset of motor symptoms (3). Patients with BSP show an increased somatosensory temporal discrimination threshold (STDT) (4). The increased STDT values remain unmodified with worsened disease severity (5). Sensory tricks, also called “geste antagoniste,” are voluntary maneuvers that alleviate the severity of abnormal movement or postures in dystonia patients (6). Sensory tricks are a cardinal feature of many forms of focal dystonia, especially common in cervical dystonia but also present in BSP, oromandibular dystonia, and writer's cramp (7). According to a survey, sensory tricks can occur in up to 71.2% of patients with BSP (8). A more frequently reported trick is using the index finger and/or thumb to touch the upper eyelid (6). Other tricks include wearing tinted lenses, singing, talking, or chewing gum (9). In animal experiments, altering the sensory input by a peripheral injury can elicit involuntary blinking and eyelid spasms in predisposed animals (10), further indicating the regulatory role of the sensory system in BSP.

The pathophysiological mechanisms of BSP are not clear. Alterations of synaptic plasticity, including disruption of homeostatic plasticity, widespread facilitation of synaptic potentials, and loss of synaptic inhibitory processes, are currently considered endophenotypic features of focal dystonia (11, 12). Abnormal sensorimotor integration functions may be related to maladaptive plasticity phenomena, which can contribute to the co-contraction of antagonistic muscle groups involved in the onset of dystonic movements (13, 14).

The startle reflex is a rapid and involuntary motor response triggered by a sudden and intense sensory stimulus (e.g., sound, electricity, or touch) (15). The startle reflex typically manifests as an eyeblink response (blink reflex) in humans and as a whole-body motor response in animals (16, 17). Prepulse inhibition (PPI) occurs when a weak prestimulus (prepulse) 30–500 ms before the startling stimulus significantly inhibits the startle reflex (18). PPI of the blink reflex causes inhibition of the *R*_2_ magnitude, while short interstimulus intervals (ISIs) increase the amplitude of *R*_1_ (19). Prepulse inhibition is believed to be a model of sensorimotor gating across taxa (20). Numerous studies have confirmed that PPI impairment is an important feature of several psychiatric disorders, including schizophrenia and obsessive-compulsive disorder (21, 22). In recent years, PPI impairment has also been observed in movement disorders such as Parkinson's disease (23), cervical dystonia (24), and BSP (25). Previous studies have shown that PPI is most pronounced at 120 ms in healthy populations (26, 27), but no studies have been conducted in healthy populations and patients with dystonia in China or Asia.

Therefore, in the present study, we analyzed PPI at different ISIs in healthy Chinese populations, compared PPI impairment characteristics between BSP patients and healthy controls, and examined the correlations of PPI impairment with disease duration and motor symptom severity in BSP patients. We aimed to elucidate the neurophysiological mechanisms of sensorimotor gating impairment in BSP patients and to provide an objective basis for identifying biological markers, guiding treatment, and evaluating the prognosis.

## 2. Materials and methods

### 2.1. Subjects

We included 30 consecutive patients with BSP who were seen in our movement disorders clinic at the First Affiliated Hospital of Dalian Medical University and 20 gender- and age-matched healthy controls (HCs). The study was approved by the ethics committee of the First Affiliated Hospital of Dalian Medical University [identification number: PJ-KS-KY-2022-134(X)], and all subjects signed informed consent.

BSP patients met the diagnostic criteria of the Benign Essential Blepharospasm Research Foundation (BEBRF) (1). They have never received botulinum toxin injections or at least 3 months since their last botulinum toxin administration. We excluded patients with comorbidities known to affect PPI, including schizophrenia spectrum disorders and temporal lobe epilepsy with psychosis, and patients who have taken medications that affect PPI, such as dopamine receptor agonists (28).

A structured interview was conducted with all subjects to obtain their medical history, family history, and current medication and to record contraceptive use and menstrual cycle in females (29). All subjects were asked to avoid smoking or consuming caffeinated beverages at least 3 h before the experiment. In addition, all BSP patients completed the Jankovic Rating Scale (JRS) (30) to assess their motor symptom severity.

### 2.2. Methods

We used surface electromyography (EMG; Synergy, CareFusion, London, UK) to perform the electrophysiological recordings. Bandpass filters for EMG recordings were 30–3,000 Hz, and the sampling rate for signal storage was 2,000 Hz. Subjects were informed of the different types of stimuli they would receive before the experiment, but the researcher and equipment were out of their view to ensure they could not see the type of stimuli.

#### 2.2.1. Blink reflex (baseline trials)

The subjects were examined in a comfortable supine position and instructed to keep their eyes gently closed. The EMG activity of the orbicularis oculi muscle was recorded by attaching surface electrodes to the subject's skin using a conductive electrode gel. The active electrodes were placed on the lower eyelids, the reference electrodes were placed 2 cm lateral to the outer canthi, and the grounding electrode was placed on the wrist of the left upper limb. Each blink reflex was evoked by electrical stimulation (constant current rectangular pulses with a stimulation duration of 0.2 ms) above the right supraorbital notch percutaneously. The stimulus intensity was 10 times the sensory threshold, defined as the minimum stimulation intensity at which the subject could perceive at least four of eight stimuli.

#### 2.2.2. Prepulse inhibition (prepulse trials)

Prepulse inhibition was assessed by applying a prepulse stimulus at ISIs of 120 ms (PPI_120_), 200 ms (PPI_200_), and 300 ms (PPI_300_) before the supraorbital nerve stimulation. The choice of ISIs was based on previous studies (18, 25, 26). The prepulse stimuli (constant current rectangular pulses with a stimulation duration of 0.2 ms) were delivered through ring electrodes attached to the middle and distal phalanges of the right index finger at an intensity two times the sensory threshold.

Four blink reflexes were obtained in each trial. Baseline and prepulse trials were randomly mixed with a 15–25 s interval between every two trials.

## 3. Statistical analysis

Trials with artifacts were excluded. In each trial, we identified the ipsilateral *R*_1_, *R*_2_, and the contralateral *R*_2c_ of the blink reflex.

We used the area under the curve to represent the magnitude of the *R*_2_ and *R*_2c_ components of each blink reflex. Following the baseline and prepulse trials, we recorded the *R*_1_ latency and peak-to-peak amplitude as well as the bilateral *R*_2_ latencies and areas under the curve. The percentage change in *R*_2_ area was the magnitude of the PPI effect (hereafter, PPI size), and the formula was PPI size (in %) = [1 – R_2_ area at prepulse trials (120, 200, or 300 ms)/*R*_2_ area at baseline trials] × 100%.

Data analysis was performed using SPSS 25.0 (SPSS, Chicago, IL, USA). The normality of data was tested using the Shapiro-Wilk test. Age, gender, and sensory thresholds for supraorbital nerve stimulation and prepulse stimulus were compared between the BSP and HC groups using the independent-sample *t*-test for quantitative data and Mann-Whitney *U*-test for qualitative data and non-normally distributed data. The presence of sensory tricks was expressed as a percentage of the total number of BSP patients. Disease duration and JRS score were presented as the mean ± standard deviation (SD).

We compared PPI size at different ISIs in HCs using the one-way analysis of variance. The ipsilateral *R*_1_ latency and amplitude, bilateral *R*_2_ latencies and areas at baseline and prepulse trials, and PPI size at different ISIs were compared separately between BSP patients and HCs with the Mann-Whitney *U*-test for non-normally distributed data, and the independent-sample *t*-test for normally distributed data. We also used the Wilcoxon rank-sum test for non-normally distributed data and a paired-sample *t*-test for normally distributed data to compare within-group differences in the ipsilateral *R*_1_ latency and amplitude, bilateral *R*_2_ latencies and areas at baseline and prepulse trials.

We further divided BSP patients into those with sensory tricks (ST group) and those without sensory tricks (NST group) and compared the PPI size of two subgroups. All data obtained above were expressed as the mean ± SD. The correlations of PPI size with disease duration and JRS score in the BSP group were analyzed with Pearson correlation analysis.

All *p*-values < 0.05 was considered a significant difference.

## 4. Results

### 4.1. Clinical data

There were no significant differences between BSP and HC groups regarding age, gender, or sensory thresholds for supraorbital nerve stimulation and prepulse stimulus to the index finger (Table 1). Sensory tricks were present in 17 patients. No significant differences were found in the clinical characteristics and demographics between ST and NST groups (Table 2).

**Table 1:** Demographic and clinical characteristics of the HC and BSP groups.

	**HC (*n* = 20)**	**BSP (*n* = 30)**	***p***
Age (years)	52.7 ± 12.6	59.4 ± 12.8	0.071
Gender M/F *n* (%)	7/13 (35%/65%)	11/19 (36.7%/63.3%)	0.904
Supraorbital threshold (mA)	1.4 ± 0.3	1.5 ± 0.3	0.210
Index finger threshold (mA)	1.7 ± 0.3	1.9 ± 0.7	0.380
Duration (years)	-	4.8 ± 3.1	-
JRS score	-	5.0 ± 1.6	-
Sensory trick *n* (%)	-	17 (56.7%)	-

Non-normally distributed and qualitative data were analyzed using the Mann-Whitney U-test, and normally distributed data were analyzed using the independent-sample t-test.

**Table 2:** Demographic and clinical characteristics of the NST and ST groups.

	**NST (*n* = 13)**	**ST (*n* = 17)**	***p***
Age (years)	61.3 ± 11.3	58.0 ± 12.7	0.497
Gender M/F *n* (%)	4/9 (30.8/69.2%)	7/10 (41.2/58.8%)	0.558
Supraorbital threshold (mA)	1.5 ± 0.2	1.5 ± 0.4	0.527
Index fingers threshold (mA)	2.0 ± 1.8	1.7 ± 0.5	0.247
Duration (years)	4.4 ± 2.7	5.1 ± 3.6	0.720
JRS score	4.9 ± 1.3	5.1 ± 1.8	0.949

Non-normally distributed and qualitative data were analyzed using the Mann-Whitney U-test, and normally distributed data were analyzed using the independent-sample t-test.

### 4.2. PPI difference between the BSP and HC groups

**Figure 1:**
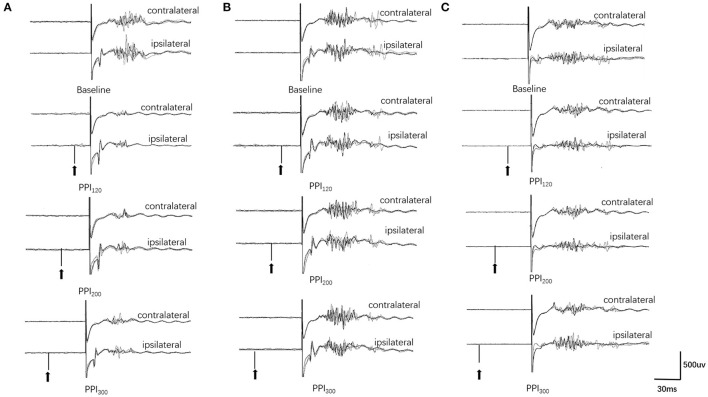
Prepulse inhibition of the blink reflex in the HC and BSP groups (with and without sensory tricks). **(A)** HC group, **(B)** NST group, **(C)** ST group. PPI_120_, prepulse inhibition at ISIs of 120 ms; PPI_200_, prepulse inhibition at ISIs of 200 ms; PPI_300_, prepulse inhibition at ISIs of 300 ms. The upper two traces represent the baseline blink reflex (without a prepulse stimulus), while the lower six traces represent the blink reflex after a prepulse stimulus (arrow indicates prepulse stimulus to the index finger). Each trace represents the superposition of four blink reflexes. BSP patients had greater *R*_2_ and *R*_2c_ areas after prepulse stimulation than HCs, and the NST group had greater *R*_2_ and *R*_2c_ areas after prepulse stimulation than the ST group (i.e., BSP patients exhibited impaired prepulse inhibition, and patients with sensory tricks showed better PPI than those without sensory tricks).

**Figure 2:**
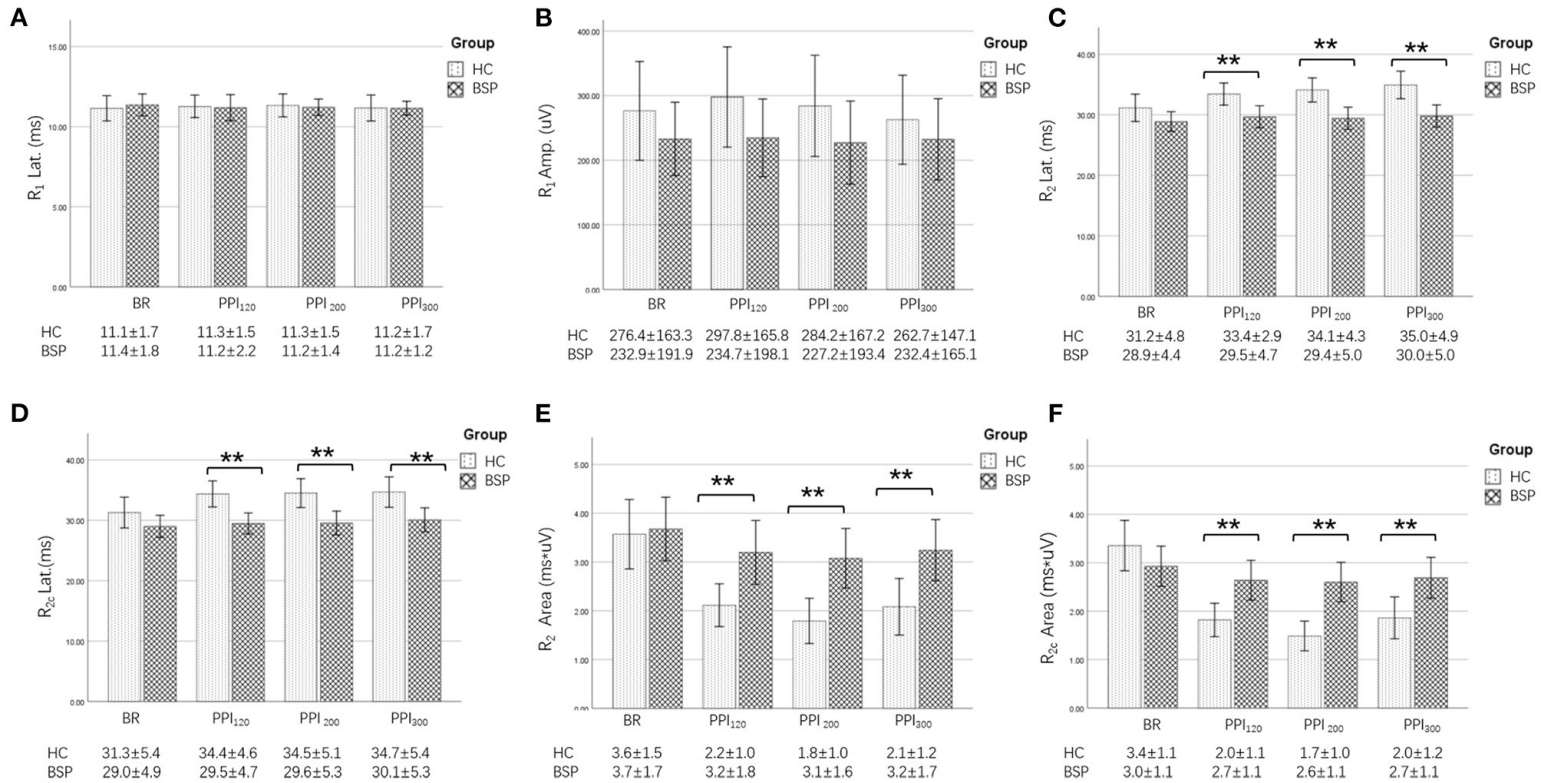
Comparison of blink-reflex neurophysiological data between the HC and the BSP groups. BR, baseline blink reflex (without a prepulse stimulus); PPI_120_, prepulse inhibition at ISIs of 120 ms; PPI_200_, prepulse inhibition at ISIs of 200 ms; PPI_300_, prepulse inhibition at ISIs of 300 ms. **(A)** Differences in the *R*_1_ latency between the BSP and HC groups at baseline and prepulse trials administered at different ISIs. **(B)** Differences in the *R*_1_ amplitude between the BSP and HC groups at baseline and prepulse trials administered at different ISIs. **(C)** Differences in the *R*_2_ latency between the BSP and HC groups at baseline and prepulse trials administered at different ISIs. **(D)** Differences in the *R*_2c_ latency between the BSP and HC groups at baseline and prepulse trials administered at different ISIs. **(E)** Differences in the *R*_2_ area between the BSP and HC groups at baseline and prepulse trials delivered at different ISIs. **(F)** Differences in the *R*_2c_ area between the BSP and HC groups at baseline and prepulse trials delivered at different ISIs. Non-normally distributed and qualitative data were analyzed using the Mann-Whitney *U*-test, and normally distributed data were analyzed using the independent-sample *t*-test. **p < 0.01.

**Figure 3:**
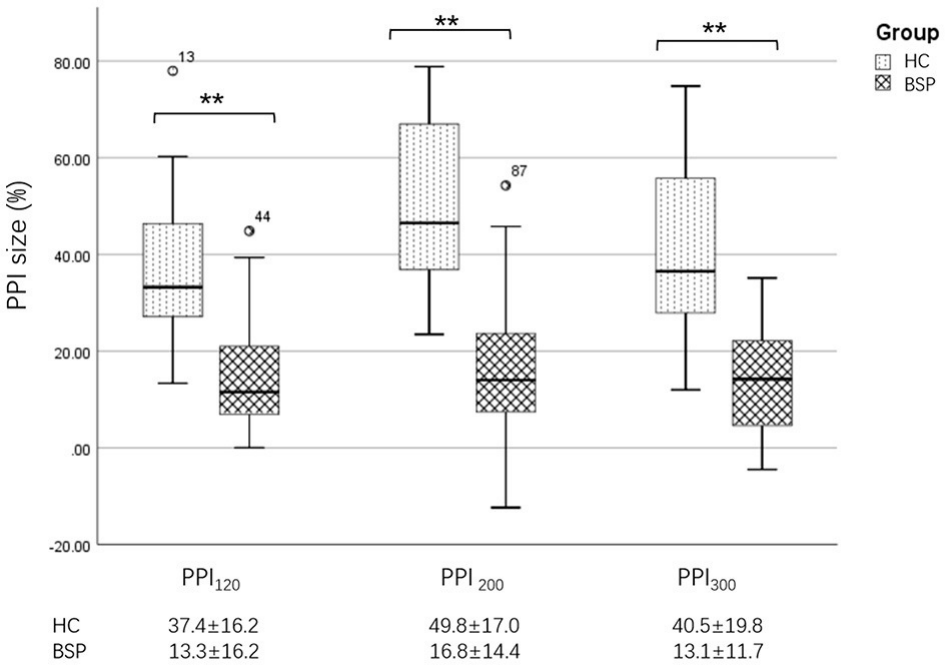
Differences in PPI size between the HC and BSP groups. PPI_120_, prepulse inhibition at ISIs of 120 ms; PPI_200_, prepulse inhibition at ISIs of 200 ms; PPI_300_, prepulse inhibition at ISIs of 300 ms. All comparisons were performed using the independent-sample *t*-test. ^**^p < 0.01.

**Figure 4:**
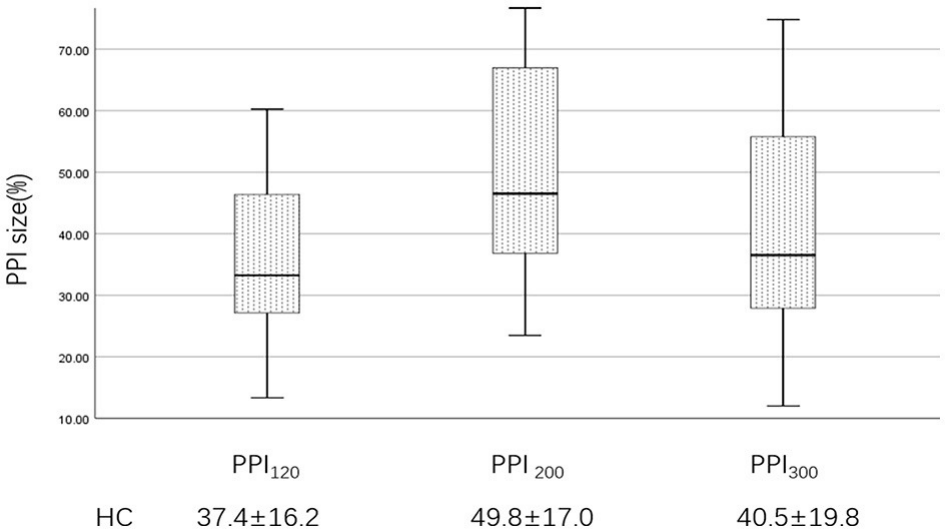
PPI size of the HC group at ISIs of 120, 200, and 300 ms. PPI_120_, prepulse inhibition at ISIs of 120 ms; PPI_200_, prepulse inhibition at ISIs of 200 ms; PPI_300_, prepulse inhibition at ISIs of 300 ms. The comparisons were performed using the one-way analysis of variance.

Examples of the blink reflex responses without and with prepulse stimulus in the BSP and HC groups are displayed in Figure 1. The characteristics of the baseline blink reflex induced by supraorbital nerve stimulation were not significantly different between the two groups (Figure 2). In the HC group, prepulse stimulus elicited bilateral *R*_2_ latencies prolongation and bilateral *R*_2_ areas reduction in all three ISIs. However, in the BSP group, prepulse stimulus had no significant effect on bilateral *R*_2_ latencies, and the inhibition of bilateral *R*_2_ areas was lower than that in the HC group. That is, the PPI size in the BSP group was significantly smaller than that in the HC group (Table 3; Figure 3). In addition, the prepulse stimulus increased the *R*_1_ amplitude at ISIs of 120 ms in HCs, which was absent in the BSP group (Table 3). Besides, we performed a correlation analysis between PPI and age in the HC and BSP groups but found no significant correlation (the date was not shown). Although there was no significant difference, the PPI size appeared greater at 200 ms compared to 120 ms and 300 ms in HCs (Figure 4).

**Table 3:** Within-group neurophysiological differences in the blink reflex at baseline and prepulse trials.

	**HC**							**BSP**						
	**Baseline**	**PPI**_120_	***p***	**PPI**_200_	***p***	**PPI**_300_	***p***	**Baseline**	**PPI**_120_	***p***	**PPI**_200_	***p***	**PPI**_300_	***p***
*R*_1_ Lat.	11.1 ± 1.7	11.3 ± 1.5	0.600	11.3 ± 1.5	0.300	11.2 ± 1.7	0.862	11.4 ± 1.8	11.2 ± 2.2	0.312	11.2 ± 1.4	0.735	11.2 ± 1.2	0.672
*R*_1_ Amp.	276.4 ± 163.3	297.8 ± 165.8	0.042^*^	284.2 ± 167.2	0.632	262.7 ± 147.1	0.103	232.9 ± 191.9	234.7 ± 198.1	0.992	227.2 ± 193.4	0.349	232.4 ± 165.1	0.894
*R*_2_ Lat.	31.2 ± 4.8	33.4 ± 2.9	0.004^**^	34.1 ± 4.3	0.000^**^	35.0 ± 4.9	0.000^**^	28.9 ± 4.4	29.5 ± 4.7	0.119	29.4 ± 5.0	0.364	30.0 ± 5.0	0.136
*R*_2c_ Lat.	31.3 ± 5.4	34.4 ± 4.6	0.003^**^	34.5 ± 5.1	0.001^**^	34.7 ± 5.4	0.003^**^	29.0 ± 4.9	29.5 ± 4.7	0.621	29.6 ± 5.3	0.491	30.1 ± 5.3	0.060
*R*_2_ Area	3.6 ± 1.5	2.2 ± 1.0	0.000^**^	1.8 ± 1.0	0.000^**^	2.1 ± 1.2	0.000^**^	3.7 ± 1.7	3.2 ± 1.8	0.000^**^	3.1 ± 1.6	0.000^**^	3.2 ± 1.7	0.000^**^
*R*_2c_ Area	3.4 ± 1.1	2.0 ± 1.1	0.000^**^	1.7 ± 1.0	0.000^**^	2.0 ± 1.2	0.000^**^	3.0 ± 1.1	2.7 ± 1.1	0.001^**^	2.6 ± 1.1	0.001^**^	2.7 ± 1.1	0.003^**^

PPI_120_, prepulse inhibition at ISIs of 120 ms; PPI_200_, prepulse inhibition at ISIs of 200 ms; PPI_300_, prepulse inhibition at ISIs of 300 ms; Lat., latency; Amp., amplitude.

Non-normally distributed data were analyzed using the Wilcoxon rank-sum test, and normally distributed data were analyzed using the paired-sample t-test.

^*^p < 0.05; ^**^p < 0.01.

### 4.3. PPI difference between the ST and NST groups

**Figure 5:**
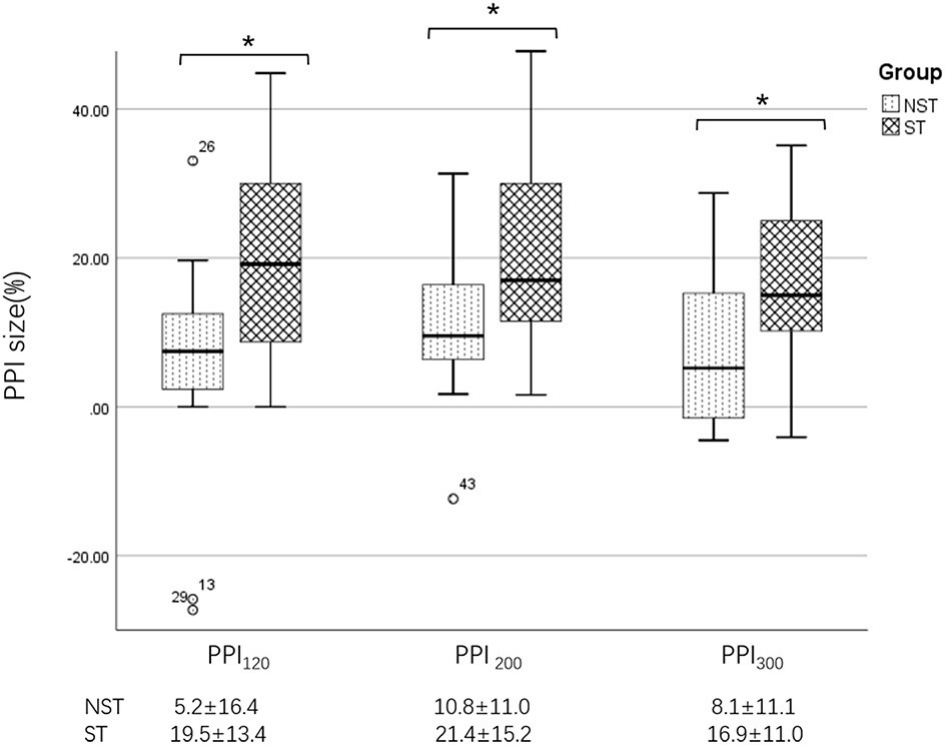
Differences in PPI size between the ST and NST groups. PPI_120_, prepulse inhibition at ISIs of 120 ms; PPI_200_, prepulse inhibition at ISIs of 200 ms; PPI_300_, prepulse inhibition at ISIs of 300 ms. All comparisons were performed using the independent-sample *t*-test. *p < 0.05.

Further analysis revealed that, in BSP patients, the PPI size was significantly greater in the ST group than in the NST group (Figures 1, 5). Finally, we found no correlations between PPI size and motor symptom severity or disease duration.

## 5. Discussion

To our knowledge, this is the first study of PPI impairment in BSP patients from a Chinese population. Previous European studies have shown that PPI size in healthy populations is generally above 60% (24, 31). In contrast, the present study reported a smaller PPI of about 40%. Additionally, previous studies have shown that PPI is most pronounced at 120 ms in healthy populations (26, 27), but in our research, PPI size appeared to be greater at 200 ms. The differences in ethnicity may contribute to the discrepancy of results, in addition to age and gender structures (27, 32, 33). Furthermore, we found that impaired PPI in BSP patients also occurred at ISIs of 200 and 300 ms, and the *R*_1_ amplitude facilitation and bilateral *R*_2_ latencies prolongation were absent in BSP patients, which above was not explored in previous studies (25, 34).

A supraorbital nerve stimulation induces two primary responses in the orbicularis oculi: an early ipsilateral component (*R*_1_) and a late bilateral component (*R*_2_) (35). The *R*_1_ originates from an oligosynaptic pontine circuit, while the *R*_2_ is mediated by multisynaptic pontomedullary connections (36). The main anatomical structures responsible for PPI are located in the brainstem, as PPI can still be observed in decerebrate animals (37). The central structure may be the connection between the pedunculopontine tegmental nucleus (PPTN) and the caudal pontine reticular nucleus (38). Although PPI occurs in the brainstem, it is also subject to top-down modulation by forebrain regions (39, 40). In the present study, the unconditioned blink reflex did not differ between BSP patients and HCs, indicating the integrity of the brainstem circuits. Abnormal top-down modulation from the prefrontal lobe projections to the pontine reflex circuits may be the reason for impaired PPI. Previous studies have found an abnormal blink reflex recovery cycle in BSP patients, confirming hyperexcitability of the trigemino-facial circuits (41). In our study, impaired PPI in BSP patients suggests abnormal inhibitory modulation at the cortical and subcortical levels may also contribute to the hyperexcitability of the trigemino-facial circuits.

Early animal studies have identified a cortico-subcortical pathway from the brainstem that mediates PPI, called the cortico-pallidum-thalamic (CSPT) circuit (42). Neuroimaging studies demonstrated the involvement of the frontal and parietal cortical regions, striatum, hippocampus and thalamus in PPI (43). In recent years, studies have reported abnormal activation and functional connectivity in the frontal and parietal cortex, basal ganglia, and cerebellar in BSP (44). In addition, cerebellar continuous theta burst stimulation can improve motor symptoms in patients with dystonia (45, 46). It has been suggested that PPTN had a reciprocal association with basal ganglia (47). The PPTN also participated in muscle tone regulation (48). Therefore, PPTN may play a role in the dysregulation of PPI in BSP. Our previous studies also showed abnormal functional connectivity of sensorimotor networks and regulatory networks involving the frontal lobe in BSP (49). Thus, impairment of central nervous system inhibition may lead to excessive motor output, and abnormal cortical and subcortical regulation may also contribute to the abnormal PPI in BSP.

We demonstrated that PPI impairment is greater in BSP patients without sensory tricks, similar to the results of previous studies (25). Gomez-Wong et al. found that the *R*_2_ magnitude of the blink reflex was reduced when a sensory trick was induced by a light touch on the eyelids and periorbital areas of the face in BSP patients, suggesting that sensory tricks may serve as a prepulse stimulus to modulate the activity of the trigeminal-facial circuits and thus ameliorate spasm (50). According to the “sensory-motor integration” theory, the abnormal excitation in the primary motor cortex may be due to excessive signal afferents in the primary somatosensory cortex, a phenomenon known as “sensory overflow (51).” PPI may inhibit sensory information overload through sensorimotor gating mechanisms (20). Our previous functional magnetic resonance imaging study reported a relatively preserved function of the supplementary motor in BSP patients with sensory tricks (49). Thus, the relatively normal PPI in BSP patients with sensory tricks reflects the relative preservation of sensory information processing and the ability to regulate abnormal trigemino-facial circuits excitability.

Additionally, we showed that PPI was not associated with the severity or duration of BSP, indicating abnormal PPI may represent a premorbid feature. Such PPI impairment was found not only in BSP but in cervical dystonia (24), thus may be a potential pathogenesis of dystonia.

Although we found that PPI seemed to be more pronounced at 200 ms, there was no significant difference. Besides, our study has some other limitations. We failed to explore PPI at ISIs lower than 100 ms due to equipment limitations. Considering the small sample size, we were unable to provide a subgroup analysis according to the variability of clinical symptoms, such as increased blinking and apraxia of eyelids opening.

## 6. Conclusion

In conclusion, we found that PPI was impaired in BSP patients at three different ISIs. Patients with sensory tricks had better PPI than those without sensory tricks. The sensory tricks phenomenon may represent either the relative integrity of sensorimotor gating pathways or a compensatory mechanism. We may thus speculate that the underlying pathophysiology of abnormal cortical/subcortical regulation mechanisms of sensorimotor gating and abnormal brainstem excitatory pathways may play an important role in BSP.

## Data availability statement

The raw data supporting the conclusions of this article will be made available by the authors, without undue reservation.

## Ethics statement

The studies involving human participants were reviewed and approved by the Ethics Committee of the First Affiliated Hospital of Dalian Medical University [identification number: PJ-KS-KY-2022-134(X)]. The patients/participants provided their written informed consent to participate in this study.

## Author contributions

ZL and CS were responsible for recruiting and screening eligible subjects. XY and HYW completed the study registration and obtained informed consent. RL collected clinical information. XHa and XHu designed and conducted the experiment. XHa wrote the paper, designed the tables, and drew the images. All authors have made meaningful revisions to the paper.
