# Corrigendum: Elucidation of the mechanism underlying impaired sensorimotor gating in patients with primary blepharospasm using prepulse inhibition

**DOI:** 10.3389/fneur.2023.1174368

**Published:** 2023-03-16

**Authors:** Xinqing Hao, Xiaofeng Huang, Xiaoxue Yin, Hai-Yang Wang, Ren Lu, Zhanhua Liang, Chunli Song

**Affiliations:** ^1^Department of Neurology, The First Affiliated Hospital of Dalian Medical University, Dalian, China; ^2^Department of Neurology, Jining No. 1 People's Hospital, Jining, China

**Keywords:** primary blepharospasm, prepulse inhibition, blink reflex, sensory trick, sensorimotor integration

In the published article, there was an error in Figure 3 as published. For PPI_200_ the BSP value was written as “17.3 ± 14.6” but should have been “16.8 ± 14.4.” For PPI_300_ the HC value was written as “40.5 ± 20.0” but should have been “40.5 ± 19.8”. The corrected Figure 3 appears below.

**Figure 3:**
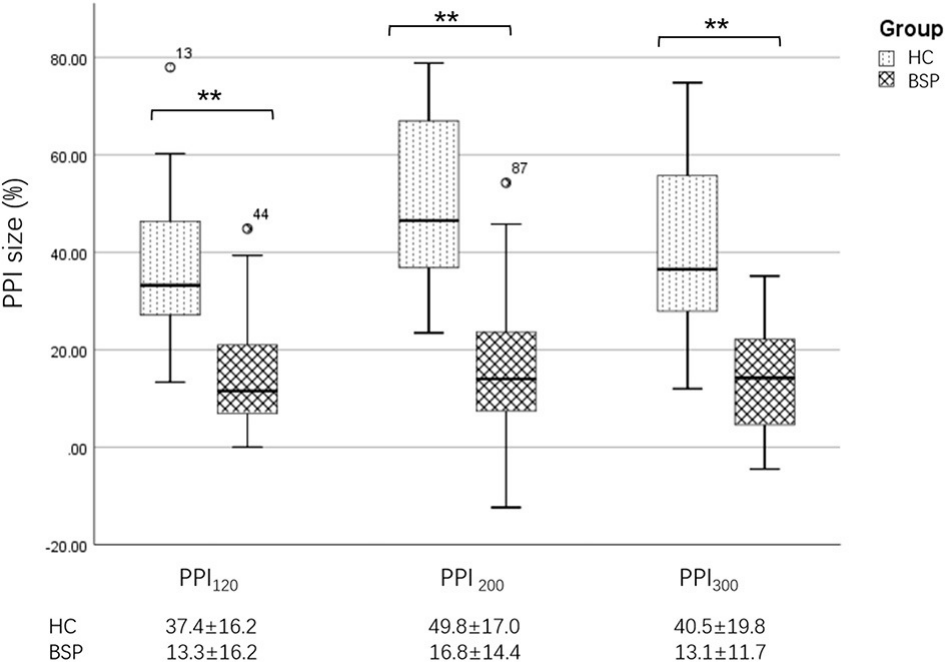
Differences in PPI size between the HC and BSP groups. PPI_120_, prepulse inhibition at ISIs of 120 ms; PPI_200_, prepulse inhibition at ISIs of 200 ms; PPI_300_, prepulse inhibition at ISIs of 300 ms. All comparisons were performed using the independent-sample *t*-test. ^**^p < 0.01.

In the published article, there were also errors in Figure 4 as published. For PPI_120_ the HC value was “39.1 ± 15.4” but should have been “37.4 ± 16.2.” For PPI_200_ the HC value was “50.1 ± 16.5” but should have been “49.8 ± 17.0.” For PPI_300_ the HC value was “41.8 ± 18.7” but should have been “40.5 ± 19.8.” The corrected Figure 4 appears below.

**Figure 4:**
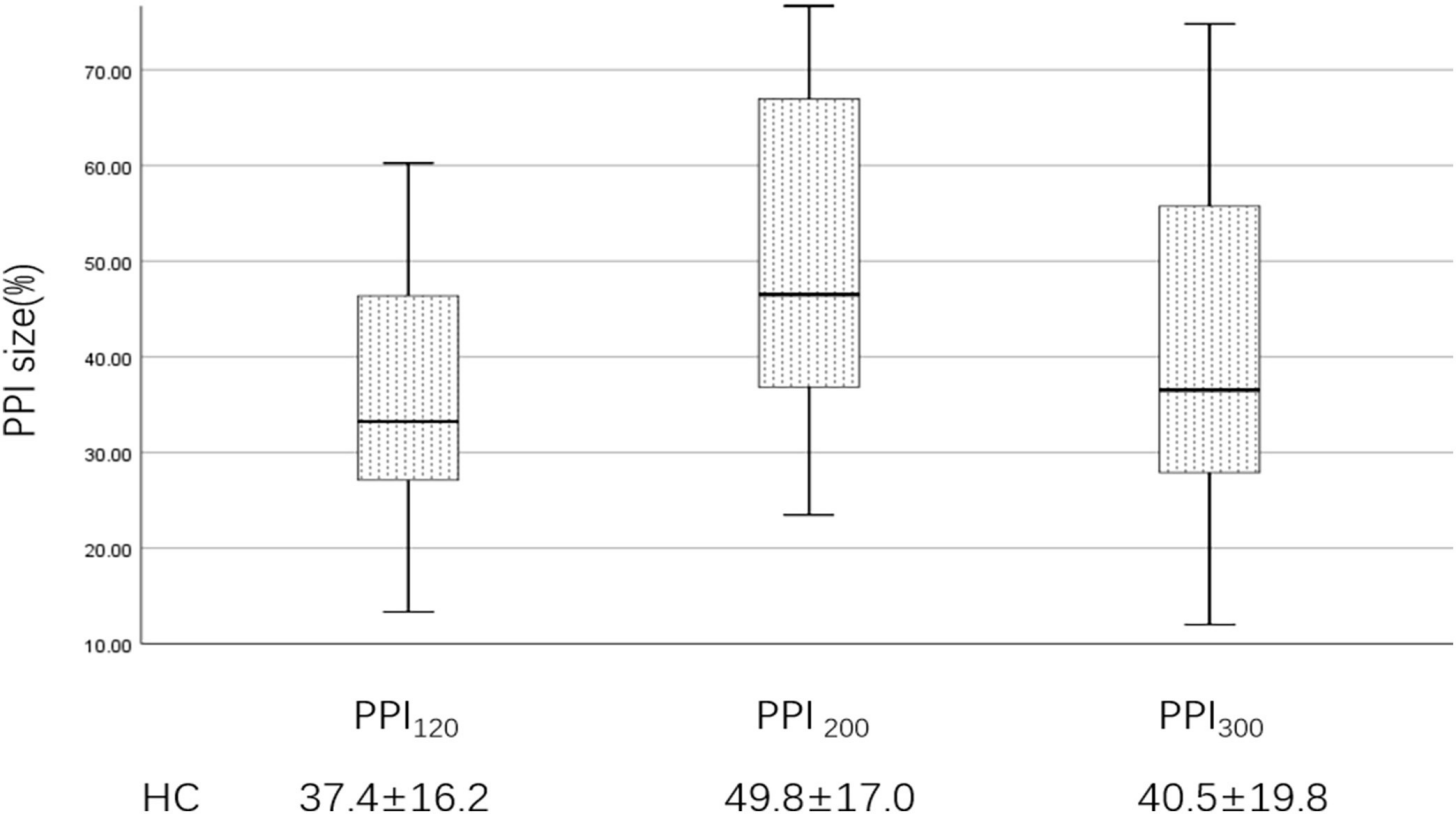
PPI size of the HC group at ISIs of 120, 200, and 300 ms. PPI_120_, prepulse inhibition at ISIs of 120 ms; PPI_200_, prepulse inhibition at ISIs of 200 ms; PPI_300_, prepulse inhibition at ISIs of 300 ms. The comparisons were performed using the one-way analysis of variance.

The authors apologize for these errors and state that this does not change the scientific conclusions of the article in any way. The original article has been updated.

